# Biologic Injection Therapy for Shoulder Disorders: A Narrative Review Comparing Platelet-Rich Plasma and Bone Marrow Aspirate Concentrate

**DOI:** 10.3390/medicina62081541

**Published:** 2026-08-11

**Authors:** Chul Hee Jung, Seok Yeon Choi, Dong Ha Lee

**Affiliations:** 1 Department of Orthopaedic Surgery, Aerospace Medical Center, Republic of Korea Air Force, Cheongju 28187, Republic of Korea; chulh2006@gmail.com; 2 Department of Rehabilitation Medicine, Aerospace Medical Center, Republic of Korea Air Force, Cheongju 28187, Republic of Korea; csyceo@naver.com; 3 Department of Orthopaedic Surgery, Seoul National University Hospital, 101 Daehak-ro, Jongno-gu, Seoul 03080, Republic of Korea

**Keywords:** shoulder disorders, rotator cuff, platelet-rich plasma, bone marrow aspirate concentrate, biologic injection, glenohumeral osteoarthritis, adhesive capsulitis, narrative review

## Abstract

*Background and Objectives*: Shoulder disorders—including rotator cuff disease, glenohumeral osteoarthritis (GH OA), and adhesive capsulitis—are among the most prevalent musculoskeletal conditions and are frequently refractory to conservative management. Platelet-rich plasma (PRP) and bone marrow aspirate concentrate (BMAC) have attracted increasing clinical interest as regenerative alternatives to corticosteroid injection, but a comparative appraisal of these two principal biologics across the full spectrum of shoulder pathology is lacking. *Materials and Methods*: This narrative review is based on structured searches of MEDLINE, Embase, Cochrane CENTRAL, and Scopus for clinical studies (randomized controlled trials [RCTs], comparative studies, and prospective series) and evidence syntheses evaluating PRP or BMAC in shoulder disorders. Studies were synthesized narratively by biologic type and disorder; no formal systematic-review or scoping-review reporting protocol was applied. Current society guidelines and consensus statements were additionally examined to position each biologic, and PRP formulation subgroups (leukocyte-rich versus leukocyte-poor) were considered. *Results*: PRP has the larger shoulder-specific evidence base, supported by several RCTs and meta-analyses in rotator cuff tendinopathy and in adhesive capsulitis; however, results are heterogeneous, effect sizes are generally modest, and some trials show no advantage over saline or corticosteroid. BMAC shoulder evidence is sparse and is strongest as a biological augment to rotator cuff repair (supported chiefly by a case-controlled study) rather than as a standalone injection; standalone shoulder BMAC RCTs are essentially absent. No published RCT directly compares PRP with BMAC for a shoulder disorder; the only direct randomized head-to-head comparison available is in knee osteoarthritis, where the two were reported to be equivalent at two years. Where PRP formulation was examined, leukocyte-poor preparations were associated with better structural and pain outcomes and leukocyte-rich preparations with functional gains in the surgical setting; current society guidance (e.g., the 2025 American Academy of Orthopaedic Surgeons [AAOS] rotator cuff guideline) does not endorse routine PRP use, and BMAC is not yet incorporated into shoulder guideline recommendations. *Conclusions*: PRP rests on a larger but still heterogeneous evidence base for shoulder disorders, whereas BMAC is biologically promising but clinically under-evidenced in the shoulder. Neither biologic is established as superior. Standardized, shoulder-specific head-to-head RCTs—incorporating biologic characterization, structural (imaging) outcomes, and longer follow-up—represent the most important research priority in this field.

## 1. Introduction

Shoulder disorders collectively represent one of the most common causes of musculoskeletal pain and functional limitation in both the general and military populations. Rotator cuff disease—encompassing tendinopathy, partial tears, and full-thickness tears—is highly prevalent, increases with age, and is a leading indication for shoulder surgery; population screening studies confirm a high prevalence of both symptomatic and asymptomatic tears [1]. Glenohumeral osteoarthritis and adhesive capsulitis further contribute substantially to shoulder-related disability. Conventional non-surgical treatments—non-steroidal anti-inflammatory drugs, physiotherapy, and intra-articular or subacromial corticosteroid injection—provide symptomatic relief but do not modify the underlying pathology, and corticosteroid exposure has been associated with adverse effects on tendon health [2,3].

Biologic injection therapies have emerged as alternatives intended to act on the tissue environment rather than to suppress inflammation alone. PRP, a centrifuged autologous blood concentrate enriched in platelets and growth factors (including PDGF, TGF-β, VEGF, EGF, and IGF-1), has been the most extensively studied biologic for shoulder pathology [4,5]. BMAC additionally provides a mesenchymal stem cell (MSC)-containing population capable of paracrine immunomodulatory and trophic signaling, with theoretical potential for tissue regeneration beyond the effects of PRP [6]. Despite growing clinical investigation, a unified comparative appraisal of PRP versus BMAC across the spectrum of shoulder disorders is absent from the literature. This narrative review addresses that gap, summarizes the clinical evidence for each biologic by disorder, compares their biological and practical profiles, and identifies priorities for future research.

To our knowledge, this is the first review to place PRP and BMAC side by side across the full range of shoulder pathology while explicitly mapping each biologic onto current society guidelines and consensus statements and comparing their molecular mediators. Its contribution is therefore not a pooled effect estimate but a structured appraisal that (i) delineates where each biologic does and does not have shoulder-specific evidence, (ii) demonstrates that the two have never been compared head-to-head in the shoulder, and (iii) translates these observations into a guideline-anchored, phenotype-based framework for clinical decision-making and a concrete research agenda.

## 2. Methods

This is a narrative review. Structured searches were performed in MEDLINE, Embase, Cochrane CENTRAL, and Scopus combining terms for biologic type (platelet-rich plasma OR PRP OR bone marrow aspirate concentrate OR BMAC OR mesenchymal stem cell injection), condition (shoulder OR rotator cuff OR glenohumeral OR adhesive capsulitis OR frozen shoulder), and study design (randomized OR controlled trial OR prospective OR systematic review OR meta-analysis). Peer-reviewed RCTs, comparative and prospective clinical studies, and evidence syntheses reporting clinical outcomes were prioritized; reference lists of relevant articles were screened for additional sources. Because current clinical practice is anchored by formal guidance, evidence-based clinical practice guidelines and society consensus statements addressing PRP or BMAC were also retrieved and appraised for the level and grade of any recommendation. Because this is a narrative rather than a systematic or scoping review, a formal reporting protocol (e.g., PRISMA) and quantitative pooling were not applied, and study selection was not exhaustive. Studies were grouped and discussed by biologic type and by shoulder disorder.

## 3. Platelet-Rich Plasma (PRP)

### 3.1. Biology and Preparation

PRP is an autologous blood-derived preparation with a platelet concentration above physiological baseline [4]. Centrifugation separates whole blood into a red-cell layer, a platelet- and leukocyte-containing buffy coat, and platelet-poor plasma. PRP is commonly classified by leukocyte content (leukocyte-rich [LR-PRP] versus leukocyte-poor [LP-PRP]) and by fibrin architecture [5]. Preparation variability across commercial systems substantially affects platelet yield, growth-factor content, and leukocyte concentration, which limits cross-study comparison; standardized preparation and reporting have therefore been formally called for [7].

### 3.2. Influence of Formulation: Leukocyte-Rich Versus Leukocyte-Poor PRP

Because “PRP” denotes a family of preparations rather than a single agent, outcomes are expected to differ by formulation, and leukocyte content is the most studied modifier. Leukocyte-poor (LP) PRP contains few white cells and is favored where a lower pro-inflammatory load is desirable, whereas leukocyte-rich (LR) PRP retains leukocytes that release additional cytokines and may drive a stronger, more inflammatory reparative response [5]. These biological differences translate into divergent clinical signals in the shoulder. In an umbrella review of eleven meta-analyses (level 1–2 evidence) of PRP in rotator cuff surgery, LP-PRP was associated with reduced retear rates, lower postoperative pain, and higher Constant scores versus non-PRP controls, but did not improve University of California, Los Angeles (UCLA), American Shoulder and Elbow Surgeons (ASES), or Simple Shoulder Test (SST) scores; LR-PRP, by contrast, improved the SST but showed no clear advantage for retear rate or Constant score [8]. The most consistent message is that leukocyte reduction favors structural healing and analgesia in the surgical setting, whereas leukocyte-rich preparations may better serve settings requiring a robust reparative stimulus. Critically, most shoulder trials still under-report platelet dose, leukocyte concentration, and activation status, so formulation-specific recommendations remain provisional and reinforce long-standing calls for standardized preparation and composition reporting [7].

### 3.3. Rotator Cuff Disease

Several RCTs have evaluated PRP for rotator cuff tendinopathy with mixed results. In a double-blind RCT, Kesikburun et al. found no significant difference between PRP and saline injection at one-year follow-up [9]. Rha et al. reported greater improvement with ultrasound-guided PRP than with dry needling in rotator cuff disease [10]. Jo et al. compared allogeneic PRP with corticosteroid injection for rotator cuff disease in a randomized trial and reported that PRP produced clinical improvement and represented a feasible alternative to corticosteroid [11]. Evidence syntheses are cautious: a systematic review of RCTs concluded that PRP could not be shown to be clearly superior for nonoperative rotator cuff disease [12], whereas a later meta-analysis reported a statistically significant but modest long-term (>24 weeks) pain benefit without a clear functional advantage [13]. A network meta-analysis of injection therapies likewise placed PRP among the more favorable options for longer-term outcomes while emphasizing between-trial heterogeneity [14]. Part of this heterogeneity likely reflects differences in PRP formulation and leukocyte content across trials (Section 3.2), which are seldom reported in a standardized way. Interest in PRP has been amplified by concern that repeated corticosteroid injection may adversely affect rotator cuff tendon health and repair [2,3]. In the surgical setting, a meta-analysis of RCTs suggested that PRP/platelet-rich fibrin augmentation of arthroscopic rotator cuff repair may improve some structural and clinical outcomes [15].

### 3.4. Adhesive Capsulitis

Evidence for PRP in adhesive capsulitis is growing but heterogeneous. A propensity-matched study of allogeneic pure PRP reported clinical benefit relative to a corticosteroid control [16], and a cohort study found single intra-articular PRP to be comparable to corticosteroid injection [17]. However, randomized evidence is not uniformly favorable: some trials and pooled analyses suggest an advantage for PRP at longer follow-up, while others find corticosteroid at least as effective during the early (freezing) phase. The mechanistic rationale—modulation of synovial inflammation and capsular fibrosis by PRP growth factors—remains plausible, but the overall evidence base is limited and inconsistent.

### 3.5. Glenohumeral Osteoarthritis

Evidence specifically addressing PRP for GH OA is scarce. Most of the orthobiologic osteoarthritis literature derives from the knee, where PRP and BMAC have been studied directly and, in a randomized comparison, were found to be equivalent at two years [18]. There is, at present, no robust placebo-controlled RCT of PRP dedicated to GH OA, and extrapolation from knee data should be made with caution given differences in joint loading, cartilage volume, and pathology (Table 1).

**Table 1:** Representative clinical studies of PRP for shoulder disorders. Findings are summarized qualitatively as reported by the original studies; consult the primary publications for full numerical results.

Study	Year	Design	Condition	Comparator	F/U	Key Finding (as Reported)
Kesikburun et al. [9]	2013	RCT (n = 40)	RC tendinopathy	Saline	12 mo	No significant difference vs. saline
Rha et al. [10]	2013	RCT (n = 39)	RC disease	Dry needling	6 mo	Greater improvement with PRP
Jo et al. [11]	2020	RCT	RC disease	Corticosteroid	≥6 mo	Improvement; feasible CS alternative
Lee et al. [16]	2021	Comparative, PSM	Adhesive capsulitis	Corticosteroid	—	Clinical benefit reported with PRP
Barman et al. [17]	2019	Cohort	Adhesive capsulitis	Corticosteroid	—	PRP comparable to corticosteroid
Lin et al. [13]	2020	SR/meta-analysis	RC tendinopathy	Mixed	>24 wk	Modest long-term pain benefit
Hurley et al. [12]	2019	Systematic review	RC disease (nonoperative)	Mixed	Varies	PRP not clearly superior

RC, rotator cuff; RCT, randomized controlled trial; SR, systematic review; PSM, propensity score matching; F/U, follow-up; CS, corticosteroid.

## 4. Bone Marrow Aspirate Concentrate (BMAC)

### 4.1. Biology and Preparation

BMAC is obtained by aspiration of bone marrow, typically from the posterior iliac crest, followed by point-of-care centrifugation to concentrate MSCs, hematopoietic progenitors, platelets, and growth factors in a single preparation [19]. Beyond a growth-factor payload, the MSC component is thought to contribute paracrine immunomodulatory and trophic signaling [6,20,21]. Importantly, the absolute number of MSCs recovered from a marrow aspirate is small; in the landmark shoulder augmentation study, the average number of MSCs returned to the patient was on the order of tens of thousands of cells [22]. The concentration and characterization of the cellular product vary widely between systems and operators, which complicates comparison across studies.

### 4.2. Rotator Cuff Augmentation

The most rigorous shoulder-specific BMAC evidence relates to surgical augmentation. In a case-controlled study, Hernigou et al. added iliac-crest bone marrow-derived MSCs to single-row rotator cuff repair in 45 patients and compared them with 45 matched controls who underwent repair without MSCs [22]. All MSC-augmented repairs had healed by six months (versus approximately two-thirds of controls), and at ten-year follow-up intact rotator cuffs were present in 87% of the MSC group versus 44% of controls; the number of transplanted cells correlated with the outcome. These data suggest that the principal demonstrated value of BMAC/MSC in the shoulder lies in augmenting surgical repair rather than as a standalone injection. Kim et al. evaluated a combined BMAC–PRP injection versus a rotator-cuff exercise program in 24 patients with partial rotator cuff tears and observed within-group clinical improvement in the injection group over three months, in a small, short-term, non-randomized comparison [23] (Table 2).

### 4.3. Glenohumeral Osteoarthritis

There is no published RCT, and essentially no shoulder-specific prospective trial, of standalone BMAC injection for GH OA. The clinical BMAC osteoarthritis literature is dominated by the knee—for example, registry and prospective data describing symptomatic improvement and a possible cell-dose relationship [24], and a randomized trial reporting that BMAC was equivalent to PRP for knee OA at two years [18]. Application of these findings to the glenohumeral joint is, at present, speculative and should not be presented as shoulder-specific evidence.

## 5. Comparative Analysis

### 5.1. Direct Head-to-Head Comparisons

A central and clinically important finding of this review is the near-complete absence of direct comparative evidence in the shoulder: no published RCT directly compares PRP with BMAC for any shoulder disorder. The only direct randomized head-to-head comparison of these two biologics in a joint comes from the knee, where BMAC and PRP were reported to be equivalent for knee osteoarthritis at two-year follow-up [18]. Consequently, any claim that one biologic is superior to the other in the shoulder is currently unsupported by direct evidence, and such statements should be avoided. This evidence gap—rather than a demonstrated equivalence—is the appropriate conclusion from the present shoulder literature.

### 5.2. Biological and Practical Comparison

PRP offers practical advantages: point-of-care preparation from peripheral venous blood, no donor-site morbidity, minimal regulatory burden, and the largest existing clinical evidence base for shoulder disorders [4,5,11,12,13,14]. BMAC adds the biological dimension of an MSC-containing product and is most compelling, on current evidence, in the context of rotator cuff repair augmentation rather than standalone injection [19,22]. BMAC harvesting requires iliac-crest aspiration with associated donor-site discomfort; this additional procedural burden must be weighed against an uncertain marginal benefit over PRP given the current lack of comparative shoulder data [25]. A qualitative comparison is summarized in Table 3.

### 5.3. Molecular Mediators Underlying PRP and BMAC

The two biologics share a core growth-factor payload but differ in the breadth of their signaling repertoire, which helps explain both their overlapping actions and BMAC’s additional theoretical potential. PRP delivers a bolus of platelet α-granule-derived growth factors—platelet-derived growth factor (PDGF), transforming growth factor-β (TGF-β), vascular endothelial growth factor (VEGF), epidermal growth factor (EGF), insulin-like growth factor-1 (IGF-1), and basic fibroblast growth factor (bFGF)—that promote cell proliferation, angiogenesis, matrix synthesis, and chemotaxis [4,6]. In leukocyte-rich formulations, co-delivered white cells add pro-inflammatory and catabolic mediators (e.g., interleukin-1β, tumor necrosis factor-α, and matrix metalloproteinases) that may aid debridement and early healing but can also amplify inflammation, whereas leukocyte-poor formulations minimize this component [5,6,8]. BMAC contains the same platelet-derived factors together with a mesenchymal stromal cell (MSC)-containing fraction whose principal contribution is paracrine: MSCs secrete immunomodulatory and trophic mediators—including interleukin-1 receptor antagonist (IL-1Ra), TGF-β, hepatocyte growth factor, VEGF, and prostaglandin E2—and release extracellular vesicles that modulate the local immune milieu and support tissue repair, rather than engrafting in large numbers [6,20,21]. BMAC is also comparatively enriched in anti-inflammatory mediators such as IL-1Ra. In practical terms, PRP acts chiefly as a concentrated growth-factor signal, whereas BMAC layers cell-directed paracrine and immunomodulatory signaling on top of that payload; whether this additional signaling yields superior shoulder outcomes is precisely the question that current shoulder-specific evidence cannot answer [6]. A qualitative summary of the principal mediators is provided in Table 4.

## 6. Positioning Within Clinical Guidelines and Consensus Statements

Situating PRP and BMAC against formal guidance clarifies their current standing and underscores the shoulder-specific evidence gap. For the rotator cuff, the most directly relevant document is the American Academy of Orthopaedic Surgeons (AAOS) evidence-based clinical practice guideline on the management of rotator cuff injuries, updated in 2025 [26]. It does not support the routine use of PRP for rotator cuff tendinopathy or partial-thickness tears—a recommendation drawn from high-quality studies—while acknowledging limited evidence that liquid PRP may reduce retear rates when used as an augment at the time of repair. The same guideline states, on the basis of strong evidence, that repeated corticosteroid injection may compromise rotator cuff integrity, consistent with the tendon-health concerns that have motivated interest in biologics [2,3]. Notably, BMAC and marrow-derived cellular therapy are not endorsed as a recommended intervention in shoulder guidelines and remain investigational.

No shoulder-specific society consensus currently governs orthobiologic use; the most developed guidance is for the knee. The ESSKA–ICRS consensus judged intra-articular PRP appropriate for knee osteoarthritis of Kellgren–Lawrence grade 0–III in patients up to 80 years of age after failure of conservative measures, but inappropriate as a first-line treatment or in end-stage (grade IV) disease [27]. European consensus work on blood-derived orthobiologics has also formalized grades of recommendation ranging from high-level scientific support to expert opinion, reflecting how unevenly the evidence is distributed. The AAOS/Orthopaedic Research Society biologics symposium similarly concluded that orthobiologics are biologically promising but that clinical evidence—and, crucially, product standardization—remains immature [25]. Taken together (Table 5), PRP appears in current guidance chiefly as a conditional or not-routinely-recommended option that is best reserved for selected scenarios, whereas BMAC has yet to accrue the shoulder-specific evidence needed to enter guideline recommendations at all. This guideline-level reading is itself part of what the present review contributes: it shows that neither biologic is endorsed as a shoulder standard of care and that BMAC, in particular, is positioned well behind PRP.

## 7. Discussion

Beyond summarizing individual studies, this review adds three elements to the existing PRP and BMAC literature: an explicit, disorder-by-disorder mapping of where each biologic is and is not supported in the shoulder; a side-by-side comparison of their molecular mediators and their standing in current guidelines; and the identification of a complete absence of head-to-head shoulder evidence as the field’s defining gap.

This review indicates that PRP is supported by a larger—though heterogeneous and only moderately convincing—clinical evidence base for shoulder disorders than BMAC. For rotator cuff tendinopathy, randomized trials range from no benefit over saline [9] to superiority over comparator interventions [10,11], and pooled analyses suggest at most a modest long-term effect [12,13,14]. For adhesive capsulitis, the evidence is mixed, with some studies favoring PRP and others favoring corticosteroid in the early phase [16,17]. For GH OA, dedicated shoulder evidence is essentially absent and is extrapolated from the knee [18,24].

BMAC shows its clearest shoulder benefit as an augment to surgical rotator cuff repair, supported principally by a case-controlled study with long follow-up [22], rather than as a standalone injection, for which shoulder-specific trials are lacking. Critically, no study directly compares PRP and BMAC in the shoulder; the only direct randomized comparison is in knee OA, where the two were equivalent [18]. The appropriate interpretation is therefore one of insufficient comparative evidence, not demonstrated equivalence or superiority.

Several methodological issues limit the field. PRP preparations are heterogeneous (LR- versus LP-PRP; variable platelet and leukocyte concentrations), and standardized characterization and reporting are needed [5,7]. BMAC studies similarly require standardized concentration and MSC characterization [19]. Clinically, disease phenotype and stage—partial versus full-thickness rotator cuff tear, radiographic grade in GH OA, and phase of adhesive capsulitis—should guide biologic selection, since the evidence is unevenly distributed across these subgroups.

From a practical standpoint, biologic selection in the shoulder can be framed by pathology, phase, and treatment goal. For refractory rotator cuff tendinopathy or low-grade partial tears in patients seeking a non-operative, low-morbidity option, PRP—preferably a well-characterized, leukocyte-defined preparation—is the more evidence-supported choice, although patients should be counseled that the expected benefit is modest and that guidelines do not endorse routine use [8,26]. In the operative setting, PRP or platelet-rich fibrin augmentation and, on more limited evidence, marrow-derived cellular augmentation may be considered to support healing and reduce retear, with leukocyte-poor PRP the better-supported formulation for structural endpoints [8,15]. For adhesive capsulitis, corticosteroid remains a reasonable and inexpensive first option in the early inflammatory phase, with PRP a plausible alternative when steroid is contraindicated or has failed [16,17]. For glenohumeral osteoarthritis, neither biologic has dedicated shoulder evidence, so any recommendation is extrapolated and should be shared explicitly with the patient. Across indications, cost, the added morbidity and procedural burden of marrow aspiration for BMAC, and the absence of head-to-head shoulder data should inform counseling; where PRP is reasonable and less invasive, the incremental value of BMAC as a standalone shoulder injection is currently unproven [25].

The field’s central need is not another narrative synthesis but primary data. Standardized, shoulder-specific studies are required, beginning with controlled experimental and preclinical work on BMAC in defined shoulder pathologies—an area the current literature almost entirely lacks—and progressing to adequately powered, head-to-head randomized trials of PRP versus BMAC that report biologic characterization (platelet dose, leukocyte content, MSC number), employ structural (imaging) as well as patient-reported endpoints, stratify by disease phenotype and stage, and extend follow-up beyond the intermediate term. Until such studies exist, comparative claims should remain cautious.

## 8. Limitations

This is a narrative review; it did not apply a formal systematic-review protocol, quantitative pooling, or formal risk-of-bias assessment, so selection bias cannot be excluded. The shoulder-specific BMAC evidence base is sparse and derives largely from a single case-controlled augmentation study and small non-randomized series. Heterogeneity in PRP preparation and outcome reporting limits comparability across studies, and durability beyond intermediate follow-up is inadequately characterized for both biologics. Finally, key osteoarthritis comparisons are available only for the knee and are extrapolated to the shoulder with caution. The guideline and formulation-subgroup data incorporated here are similarly constrained: the leukocyte-content evidence derives chiefly from the surgical-augmentation setting, and the most developed orthobiologic consensus statements are knee-specific, so their extrapolation to the shoulder is provisional.

## 9. Conclusions

For shoulder disorders, PRP rests on a larger but heterogeneous and only moderately convincing evidence base, with the most consistent signal as a nonoperative option for rotator cuff tendinopathy and as repair augmentation, and with mixed evidence in adhesive capsulitis. BMAC is biologically promising but clinically under-evidenced in the shoulder, with its strongest support as a surgical repair augment rather than a standalone injection. No direct PRP-versus-BMAC comparison exists for the shoulder, so neither biologic can be declared superior. Standardized, shoulder-specific head-to-head RCTs—incorporating biologic characterization, imaging-based structural outcomes, and longer follow-up—are the most important research priority in this field. This guideline-anchored reading reinforces that neither biologic is currently a shoulder standard of care and that dedicated experimental studies of BMAC in the shoulder, followed by standardized head-to-head randomized trials, are needed before comparative recommendations can be made.

## Figures and Tables

**Table 2:** Shoulder-specific clinical studies of BMAC. Standalone BMAC injection trials for shoulder disorders are essentially absent; the strongest data relate to surgical augmentation. Knee studies are discussed in the text as extrapolation only.

Study	Year	Design	Condition	Comparator	F/U	Key Finding (as Reported)
Hernigou et al. [22]	2014	Case-controlled (45 + 45)	RC repair augmentation	Repair without MSC	Up to 10 yr	Healing 100% vs. ~67% at 6 mo; 10-yr intact cuff 87% vs. 44%
Kim et al. [23]	2018	Prospective comparative (n = 24)	Partial RC tear (BMAC–PRP)	Exercise program	3 mo	Within-group improvement; small, short-term

RC, rotator cuff; MSC, mesenchymal stem cell; F/U, follow-up. No standalone shoulder BMAC RCT was identified.

**Table 3:** Qualitative comparison of injectables for shoulder disorders. Entries reflect general profiles and the current state of shoulder-specific evidence; they are not derived from a single quantitative source.

Parameter	PRP	BMAC	Corticosteroid
Preparation	Centrifugation of venous blood (point of care)	Centrifugation of iliac-crest marrow aspirate (point of care)	Ready to use
Harvest invasiveness	Very low (venipuncture)	Moderate (marrow aspiration)	None
Biologic content	Platelets and growth factors	Platelets, growth factors, MSC-containing fraction	Anti-inflammatory drug only
Shoulder RCT evidence	Multiple RCTs (heterogeneous)	Very limited; no standalone shoulder RCT	Extensive
Direct PRP vs. BMAC (shoulder)	None published	None published	—
Principal current role	Nonoperative trial; repair augmentation	Surgical repair augmentation	Short-term symptom control

MSC, mesenchymal stem cell; RCT, randomized controlled trial. Hyaluronic acid is also used clinically but has limited shoulder-specific evidence and is omitted here for brevity.

**Table 4:** Principal molecular mediators of PRP and BMAC and their putative roles. Entries are qualitative; presence and relative amounts vary by preparation system and are not standardized.

Mediator/Component	PRP	BMAC	Putative Role
PDGF (AA/AB/BB)	Yes	Yes	Mitogenesis, chemotaxis, angiogenesis
TGF-β	Yes	Yes	Matrix synthesis, cell differentiation, immunomodulation
VEGF	Yes	Yes	Angiogenesis
EGF/IGF-1/bFGF	Yes	Yes	Proliferation, matrix synthesis, anabolic signaling
Leukocyte-derived cytokines (IL-1β, TNF-α, MMPs)	LR-PRP: yes; LP-PRP: low	Variable	Pro-inflammatory/catabolic; debridement vs. inflammation
MSC-derived paracrine factors (IL-1Ra, HGF, PGE2, TGF-β)	No	Yes	Immunomodulation; trophic/anti-inflammatory support
Extracellular vesicles/exosomes	Minimal	Yes (MSC-derived)	Paracrine signaling, tissue repair
Nucleated/progenitor cells (MSCs, HPCs)	No	Yes	Cellular source of paracrine signaling

PDGF, platelet-derived growth factor; TGF-β, transforming growth factor-β; VEGF, vascular endothelial growth factor; EGF, epidermal growth factor; IGF-1, insulin-like growth factor-1; bFGF, basic fibroblast growth factor; IL, interleukin; TNF-α, tumor necrosis factor-α; MMP, matrix metalloproteinase; IL-1Ra, IL-1 receptor antagonist; HGF, hepatocyte growth factor; PGE2, prostaglandin E2; MSC, mesenchymal stromal cell; HPC, hematopoietic progenitor cell; LR/LP-PRP, leukocyte-rich/leukocyte-poor PRP.

**Table 5:** Position of PRP and BMAC in representative clinical guidelines and consensus statements. Knee consensus statements are included as the closest available orthobiologic guidance and are not shoulder-specific.

Guideline/Consensus (Year)	Scope	PRP Position	BMAC Position
AAOS Rotator Cuff CPG (2025) [26]	Shoulder/rotator cuff	Routine use not supported for tendinopathy or partial tears (high-quality evidence); limited evidence for retear reduction as a repair augment	Not addressed as a recommended intervention (investigational)
ESSKA–ICRS PRP consensus (2024) [27]	Knee OA	Appropriate for KL 0–III after failed conservative Tx (≤80 y); inappropriate first-line or in KL IV	Not covered (blood-derived scope)
AAOS/ORS biologics symposium (2016) [25]	Orthopaedic (general)	Promising; clinical evidence immature; standardization needed	Promising; clinical evidence immature; standardization needed

AAOS, American Academy of Orthopaedic Surgeons; CPG, clinical practice guideline; ESSKA, European Society of Sports Traumatology, Knee Surgery and Arthroscopy; ICRS, International Cartilage Regeneration and Joint Preservation Society; ORS, Orthopaedic Research Society; OA, osteoarthritis; KL, Kellgren–Lawrence; Tx, treatment. Grades of recommendation in European consensus work range from A (high-level scientific support) to D (expert opinion).

## Data Availability

No new data were created or analyzed in this study. Data sharing is not applicable to this article.
